# Supplementary figures and images for: BET family members Bdf1/2 modulate global transcription initiation and elongation in Saccharomyces cerevisiae
Source: eLife. 2021 Jun 17;10:e69619. doi: 10.7554/eLife.69619 (PMC8266393; doi:10.7554/eLife.69619)

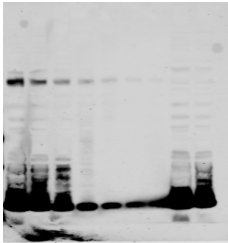

Supplement: Figure 1—figure supplement 1—source data 1. — The blot was scanned at 700/800 nm wavelengths using Li-Cor Odyssey CLx imager. The image shows results obtained for 700 nm channel. [file elife-69619-fig1-figsupp1-data1.pdf]

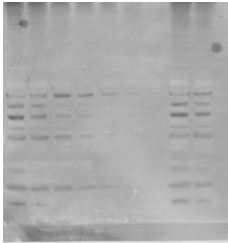

Supplement: Figure 1—figure supplement 1—source data 2. — The blot was scanned at 700/800 nm wavelengths using Li-Cor Odyssey CLx imager. The image shows results obtained for 800 nm channel. [file elife-69619-fig1-figsupp1-data2.pdf]

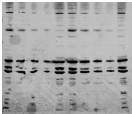

Supplement: Figure 1—figure supplement 1—source data 3. — The blot was scanned at 700/800 nm wavelengths using Li-Cor Odyssey CLx imager. The image shows results obtained for both channels. [file elife-69619-fig1-figsupp1-data3.pdf]

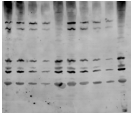

Supplement: Figure 1—figure supplement 1—source data 4. — The blot was scanned at 700/800 nm wavelengths using Li-Cor Odyssey CLx imager. The image shows results obtained for both channels. [file elife-69619-fig1-figsupp1-data4.pdf]

Figure 1-figure supplement 1-source data 5

Bdf1

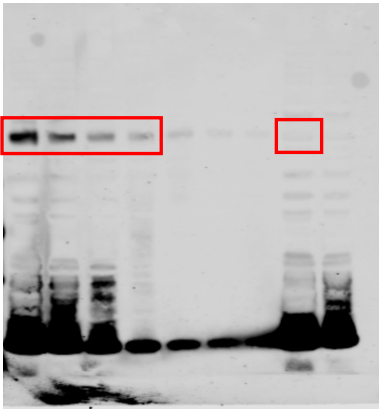

Bdf1

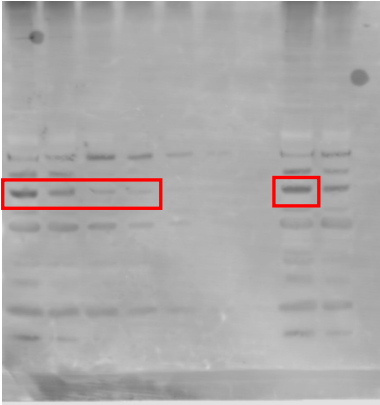

Tfg2

Bdf2

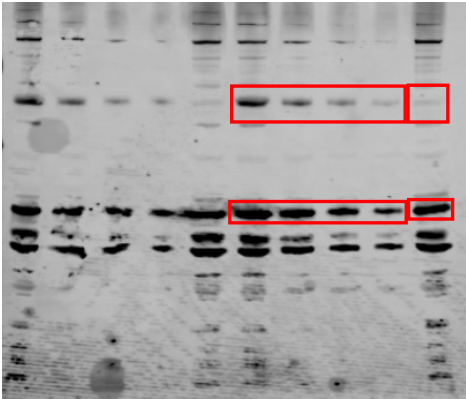

Bdf2

Tfg2

Bdf1/2

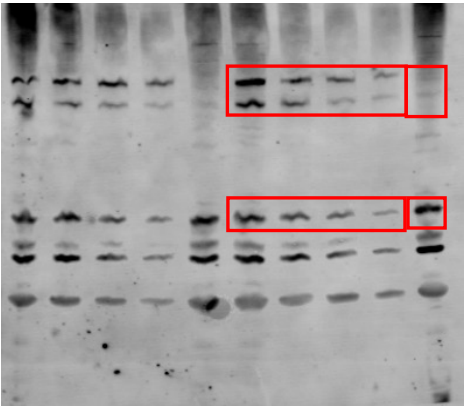

Bdf1

Bdf2

Tfg2

Supplement: Figure 1—figure supplement 1—source data 5. [file elife-69619-fig1-figsupp1-data5.pdf]

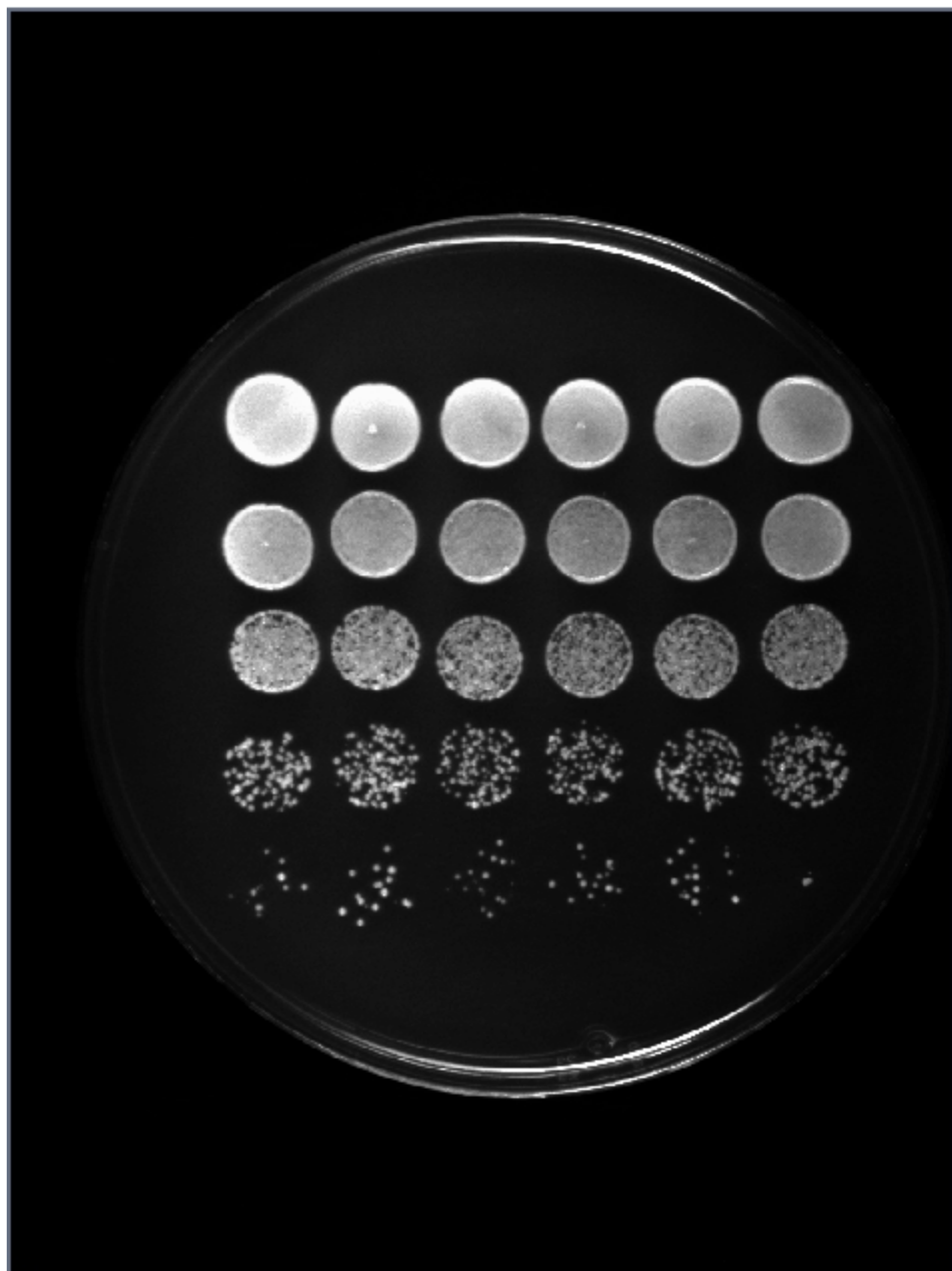

Supplement: Figure 1—figure supplement 1—source data 6. [file elife-69619-fig1-figsupp1-data6.pdf]

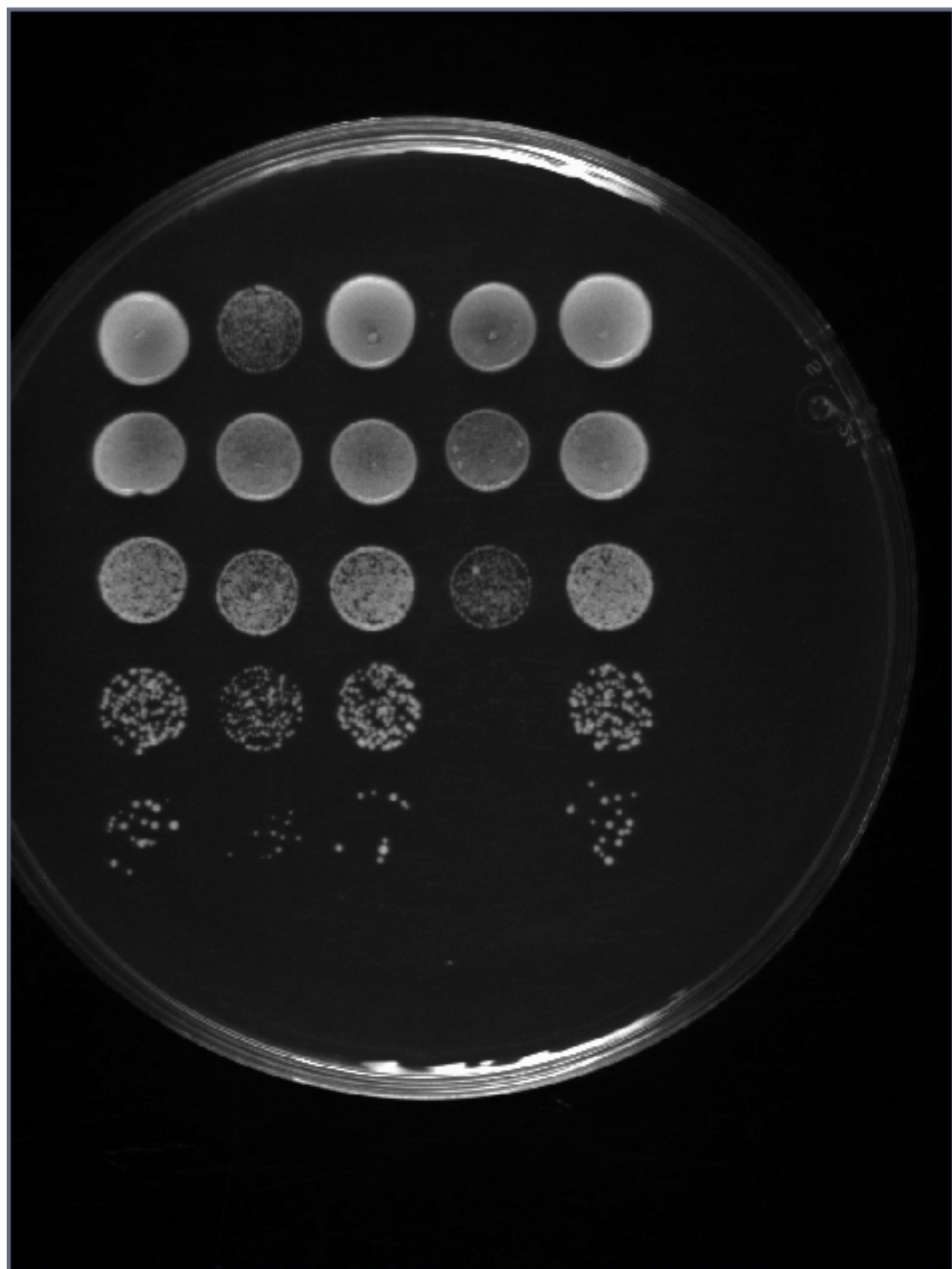

Supplement: Figure 1—figure supplement 1—source data 7. [file elife-69619-fig1-figsupp1-data7.pdf]

**Figure 1-figure supplement 1-source data 8**

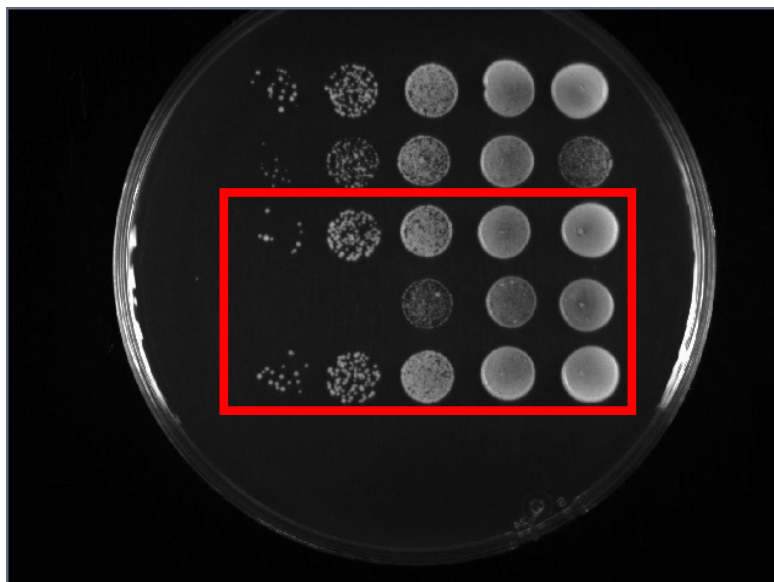

Supplement: Figure 1—figure supplement 1—source data 8. [file elife-69619-fig1-figsupp1-data8.pdf]

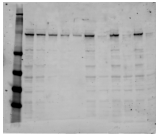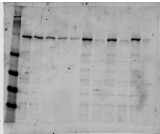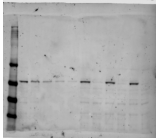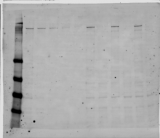

Supplement: Figure 1—figure supplement 2—source data 1. — The blot was scanned at 700/800 nm wavelengths using Li-Cor Odyssey CLx imager. The image shows results obtained for 700 nm channel. [file elife-69619-fig1-figsupp2-data1.pdf]

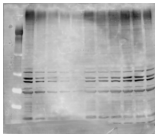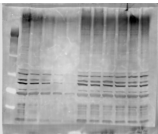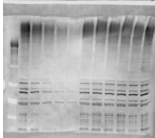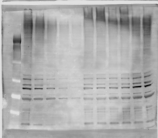

Supplement: Figure 1—figure supplement 2—source data 2. — The blot was scanned at 700/800 nm wavelengths using Li-Cor Odyssey CLx imager. The image shows results obtained for 800 nm channel. [file elife-69619-fig1-figsupp2-data2.pdf]

Figure 1–figure supplement 2–source data 3

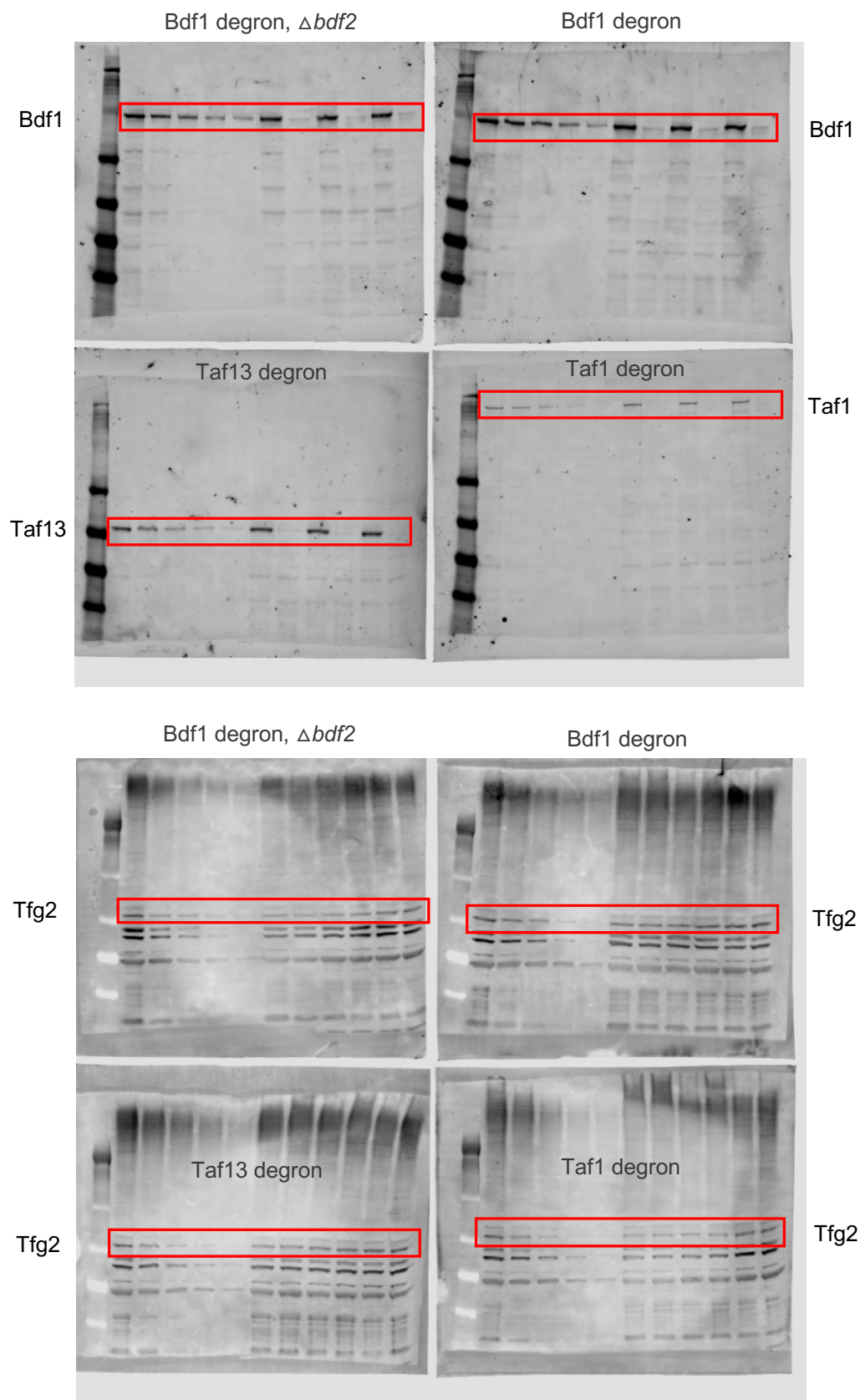

Supplement: Figure 1—figure supplement 2—source data 3. [file elife-69619-fig1-figsupp2-data3.pdf]

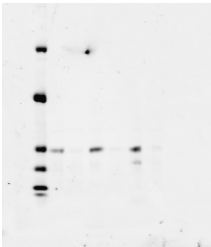

Supplement: Figure 1—figure supplement 2—source data 4. — The blot was scanned at 700 nm wavelength using Li-Cor Odyssey CLx imager. [file elife-69619-fig1-figsupp2-data4.pdf]

**Figure 1–figure supplement 2–source data 5**

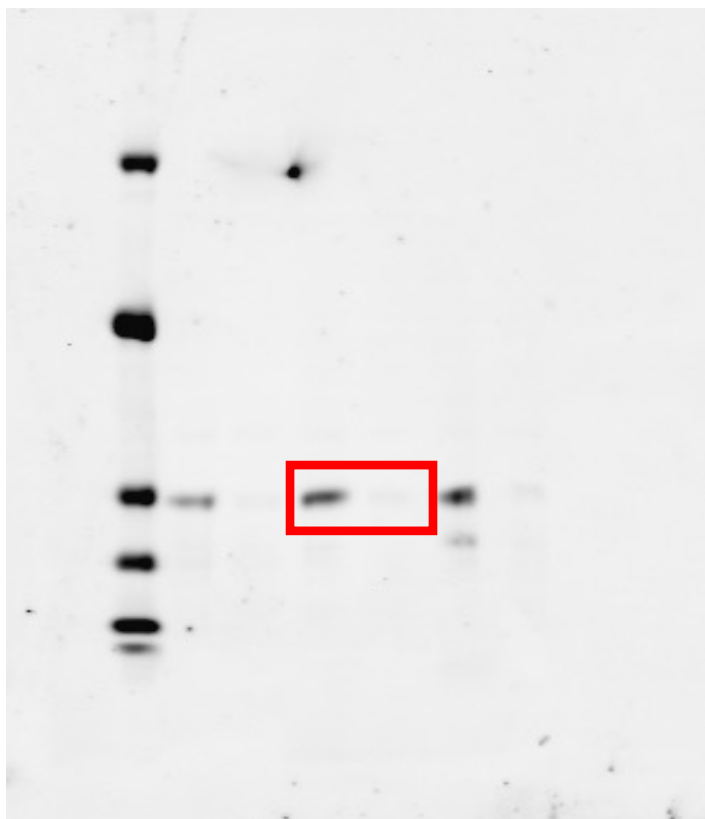

Supplement: Figure 1—figure supplement 2—source data 5. [file elife-69619-fig1-figsupp2-data5.pdf]

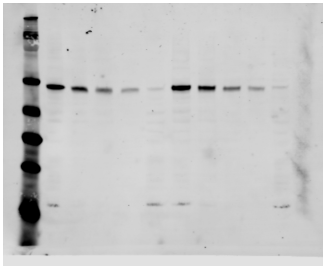

Supplement: Figure 3—figure supplement 1—source data 1. — The blot was scanned at 700/800 nm wavelengths using Li-Cor Odyssey CLx imager. The image shows results obtained for 700 nm channel. [file elife-69619-fig3-figsupp1-data1.pdf]

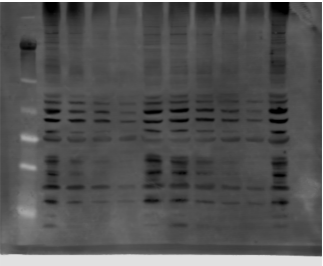

Supplement: Figure 3—figure supplement 1—source data 2. — The blot was scanned at 700/800 nm wavelengths using Li-Cor Odyssey CLx imager. The image shows results obtained for 800 nm channel. [file elife-69619-fig3-figsupp1-data2.pdf]

Figure 3—figure supplement 1—source data 3

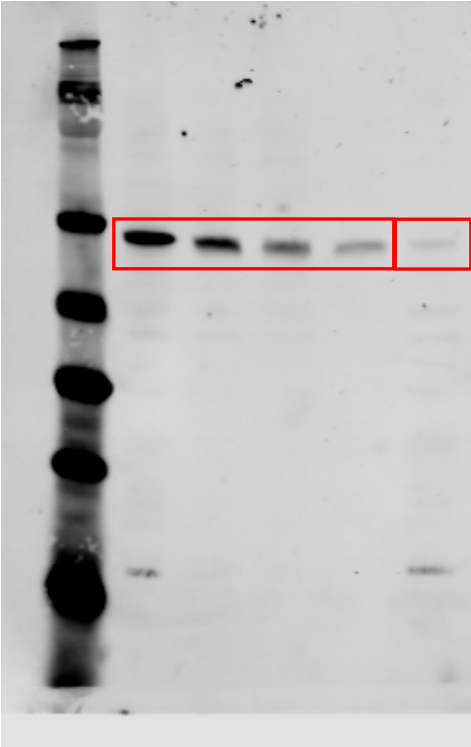

Esa1

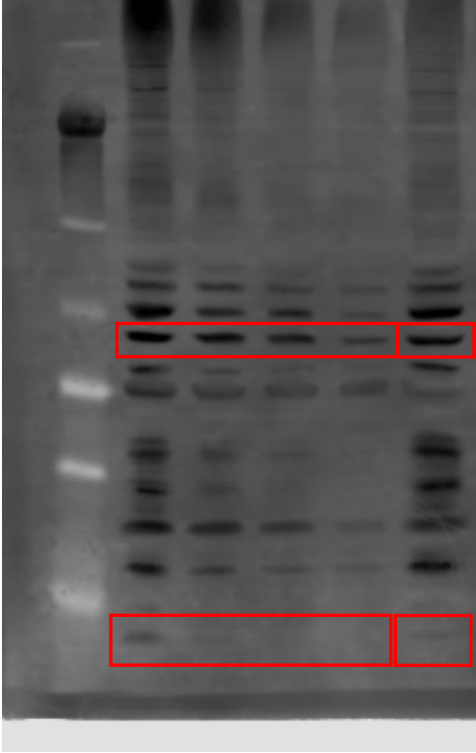

Tfg2

H4K12ac

Supplement: Figure 3—figure supplement 1—source data 3. [file elife-69619-fig3-figsupp1-data3.pdf]
