# Supplementary material for: BET family members Bdf1/2 modulate global transcription initiation and elongation in Saccharomyces cerevisiae
Source: eLife. 2021 Jun 17;10:e69619. doi: 10.7554/eLife.69619 (PMC8266393; doi:10.7554/eLife.69619)
Supplement: Transparent reporting form [file elife-69619-transrepform.docx]

***eLife’s* transparent reporting form**

We encourage authors to provide detailed information *within their submission* to facilitate the interpretation and replication of experiments. Authors can upload supporting documentation to indicate the use of appropriate reporting guidelines for health-related research (see [EQUATOR Network](http://www.equator-network.org/%20)), life science research (see the [BioSharing Information Resource](https://biosharing.org/)), or the [ARRIVE guidelines](http://www.plosbiology.org/article/info:doi/10.1371/journal.pbio.1000412) for reporting work involving animal research. Where applicable, authors should refer to any relevant reporting standards documents in this form.

If you have any questions, please consult our Journal Policies and/or contact us: [editorial@elifesciences.org](mailto:editorial@elifesciences.org).

**Sample-size estimation**

- You should state whether an appropriate sample size was computed when the study was being designed
- You should state the statistical method of sample size computation and any required assumptions
- If no explicit power analysis was used, you should describe how you decided what sample (replicate) size (number) to use

Please outline where this information can be found within the submission (e.g., sections or figure legends), or explain why this information doesn’t apply to your submission:

Selected number of biological replicates used for ChIP-seq and RNA-seq was based on expected reproducibility of experiments. Reproducibility of RNA-seq experiments was validated by analysis of distribution of coefficient of variation (Figures S1D and S4B). Reproducibility of ChIP-seq experiments was validated by detailed analysis of individual genome browser tracks and comparison of calculated changes in chromatin occupancy. In the case of the Bur1 experiment, a third biological replicate was collected. Selected ChEC-seq experiments were done in six replicates to allow for accurate estimation of the number of gene regulatory regions bound by analysed transcription factors, based on selected criteria described in the 'Materials and Methods' section. Other experiments were done in two or three biological replicates based on prior experience in mapping corresponding factors using ChEC-seq.

**Replicates**

- You should report how often each experiment was performed
- You should include a definition of biological versus technical replication
- The data obtained should be provided and sufficient information should be provided to indicate the number of independent biological and/or technical replicates
- If you encountered any outliers, you should describe how these were handled
- Criteria for exclusion/inclusion of data should be clearly stated
- High-throughput sequence data should be uploaded before submission, with a private link for reviewers provided (these are available from both GEO and ArrayExpress)

Please outline where this information can be found within the submission (e.g., sections or figure legends), or explain why this information doesn’t apply to your submission:

Number of independent biological replicates collected for each experiment is listed in the relevant paragraphs in the 'Materials and Methods' section and in Table S6. All sequencing data are deposited at Gene Expression Omnibus under accession GSE171067.

**Statistical reporting**

- Statistical analysis methods should be described and justified
- Raw data should be presented in figures whenever informative to do so (typically when N per group is less than 10)
- For each experiment, you should identify the statistical tests used, exact values of N, definitions of center, methods of multiple test correction, and dispersion and precision measures (e.g., mean, median, SD, SEM, confidence intervals; and, for the major substantive results, a measure of effect size (e.g., Pearson's r, Cohen's d)
- Report exact p-values wherever possible alongside the summary statistics and 95% confidence intervals. These should be reported for all key questions and not only when the p-value is less than 0.05.

Please outline where this information can be found within the submission (e.g., sections or figure legends), or explain why this information doesn’t apply to your submission:

All relevant information about statistical methods used is in the ‘Materials and Methods’ section and in the figure legends.

(For large datasets, or papers with a very large number of statistical tests, you may upload a single table file with tests, Ns, etc., with reference to sections in the manuscript.)

**Group allocation**

- Indicate how samples were allocated into experimental groups (in the case of clinical studies, please specify allocation to treatment method); if randomization was used, please also state if restricted randomization was applied
- Indicate if masking was used during group allocation, data collection and/or data analysis

Please outline where this information can be found within the submission (e.g., sections or figure legends), or explain why this information doesn’t apply to your submission:

Not relevant for this study.

**Additional data files (“source data”)**

- We encourage you to upload relevant additional data files, such as numerical data that are represented as a graph in a figure, or as a summary table
- Where provided, these should be in the most useful format, and they can be uploaded as “Source data” files linked to a main figure or table
- Include model definition files including the full list of parameters used
- Include code used for data analysis (e.g., R, MatLab)
- Avoid stating that data files are “available upon request”

Please indicate the figures or tables for which source data files have been provided:

Additional source data has been provided for figures S1 and S4. All other figures are based on data provided in supplementary tables S2 - S5 or, in case of line plots showing average signal, in WIG files deposited at Gene Expression Omnibus under accession GSE171067.
